# Supplementary material for: Deforestation-induced climate change reduces carbon storage in remaining tropical forests
Source: Nat Commun. 2022 Apr 12;13:1964. doi: 10.1038/s41467-022-29601-0 (PMC9005651; doi:10.1038/s41467-022-29601-0)
Supplement: Supplementary file 1 — Supplementary Information [file 41467_2022_29601_MOESM1_ESM.pdf]

Supplementary Information for

Deforestation-induced climate change reduces carbon storage in remaining tropical forests

Yue Li[1*], Paulo M. Brando[1], Douglas C. Morton[2], David M. Lawrence[3], Hui Yang[4], James T. Randerson[1]

[1]*Department of Earth System Science, University of California, Irvine, CA, USA*
[2]*Biospheric Sciences Laboratory, NASA Goddard Space Flight Center, Greenbelt, MD, USA*
[3]*National Center for Atmospheric Research, Boulder, CO, USA*
[4]*Department of Biogeochemical Integration, Max Planck Institute for Biogeochemistry, Jena, Germany*

[*]Corresponding author: yue.li@uci.edu

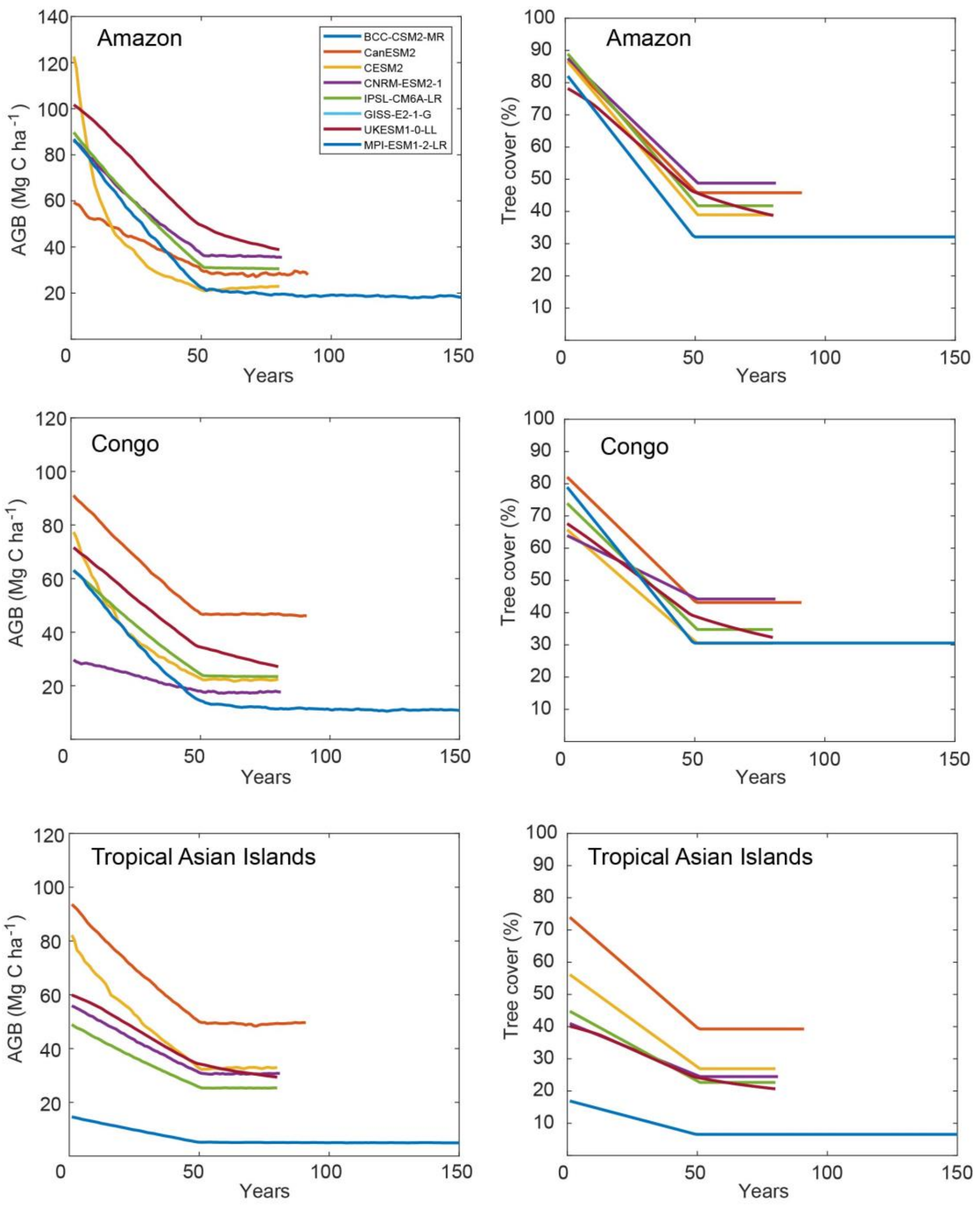

Supplementary Figure 1 | Simulated temporal changes in aboveground biomass (AGB, being converted from simulated total vegetation carbon using an empirical factor as described in Methods, unit: Mg C ha^{-1}) and tree cover fraction (%) across three major tropical forest ecosystems in LUMIP deforest–glob experiments (Table 1). Note that the AGB and tree cover fraction are not available in the output for BCC–CSM2–MR and GISS–E2–1–G.

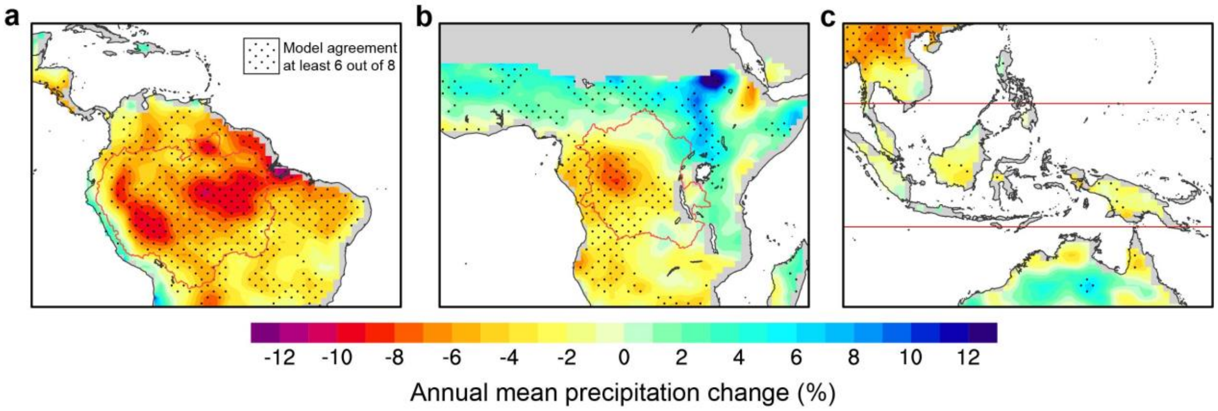

Supplementary Figure 2 | Same as Fig. 1a–c but in percentage (%) for the annual mean precipitation change in response to idealized deforestation in LUMIP experiments.

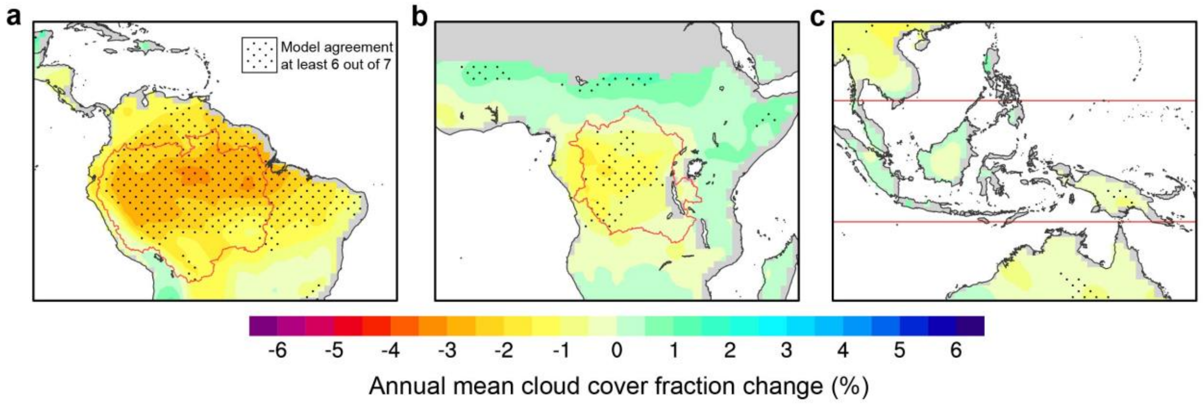

Supplementary Figure 3 | Biophysical impacts of idealized deforestation on total cloud cover fraction (%) in three tropical regions. Changes in mean annual cloud cover in **a**, South America, **b**, Africa and **c**, Southeast Asia. The changes were computed as the difference between the average of last 30 years from the LUMIP deforest–glob and piControl experiments (see Methods). Dotted area indicates the model agreement, with at least 6 models agreeing on the sign of the cloud responses. Information of the 8 models is listed in Table 1. Note that the cloud cover fraction is not available in the output for model BCC–CSM2–MR.

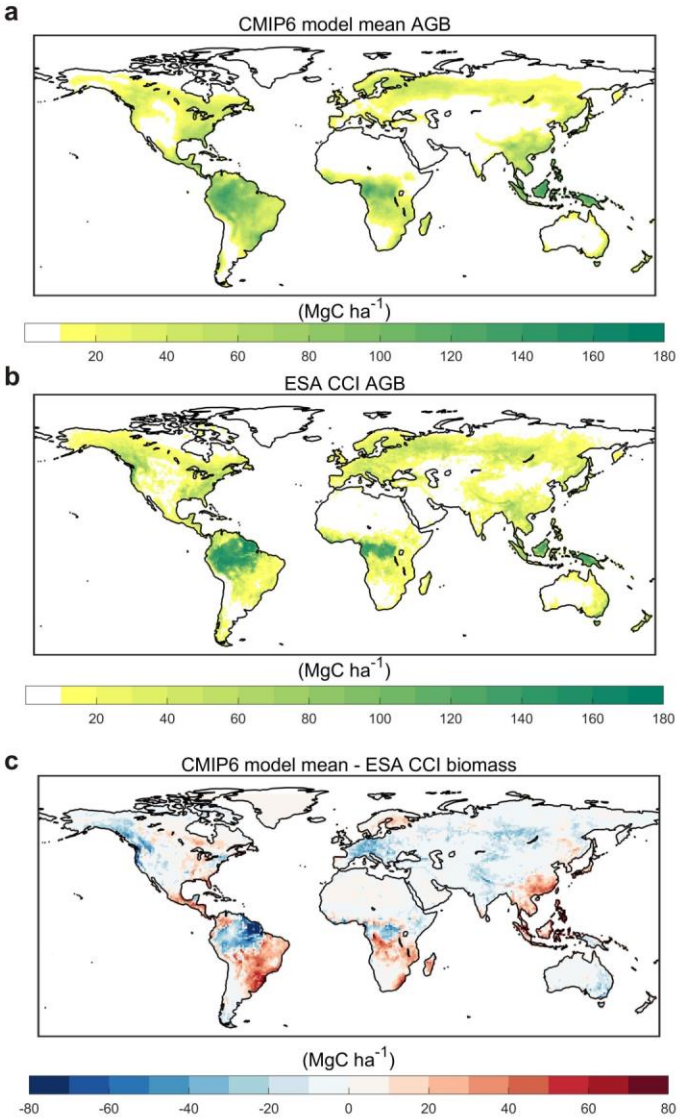

Supplementary Figure 4 | Spatial pattern of the CMIP6 model mean AGB (a) and satellite–based AGB from ESA–CCI BIOMASS project (b; see data descriptions in Methods). AGB from CMIP6 models were computed from the total vegetation carbon multiplied by a mean factor weighted by the simulated tree cover fraction (see Methods) and were averaged across models during the last 30 years in the piControl simulations. AGB from ESA–CCI corresponds to the year of 2017. Panel c shows the difference between the AGB estimates in the top two panels (panel a minus panel b).

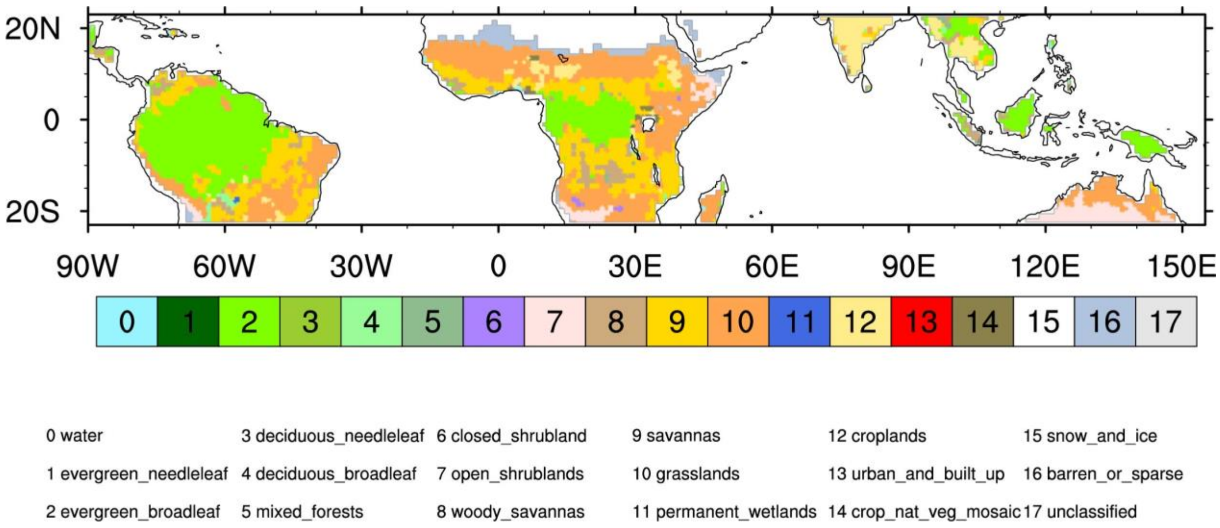

Supplementary Figure 5 | Tropical land cover map showing land cover type for each 1–degree grid cell within 23ºS–23ºN. In our analysis, desert regions with mean annual precipitation less than 100 mm yr^{-1} and grid cells with a land fraction less than 50% were masked when computing the aboveground biomass (AGB) climate sensitivity to avoid use of observations where uncertainties are high. The land cover map was derived from MODIS Land Cover Type (MCD12C1) during the year 2017 (consistent with the ESA–CCI AGB map), with the International Geosphere-Biosphere Programme (IGBP) class system as shown above. The MCD12C1 map has a spatial resolution of 0.05 degree and has been resampled into a 1–degree map with each 1–degree grid cell in the map showing the most abundant land cover type.

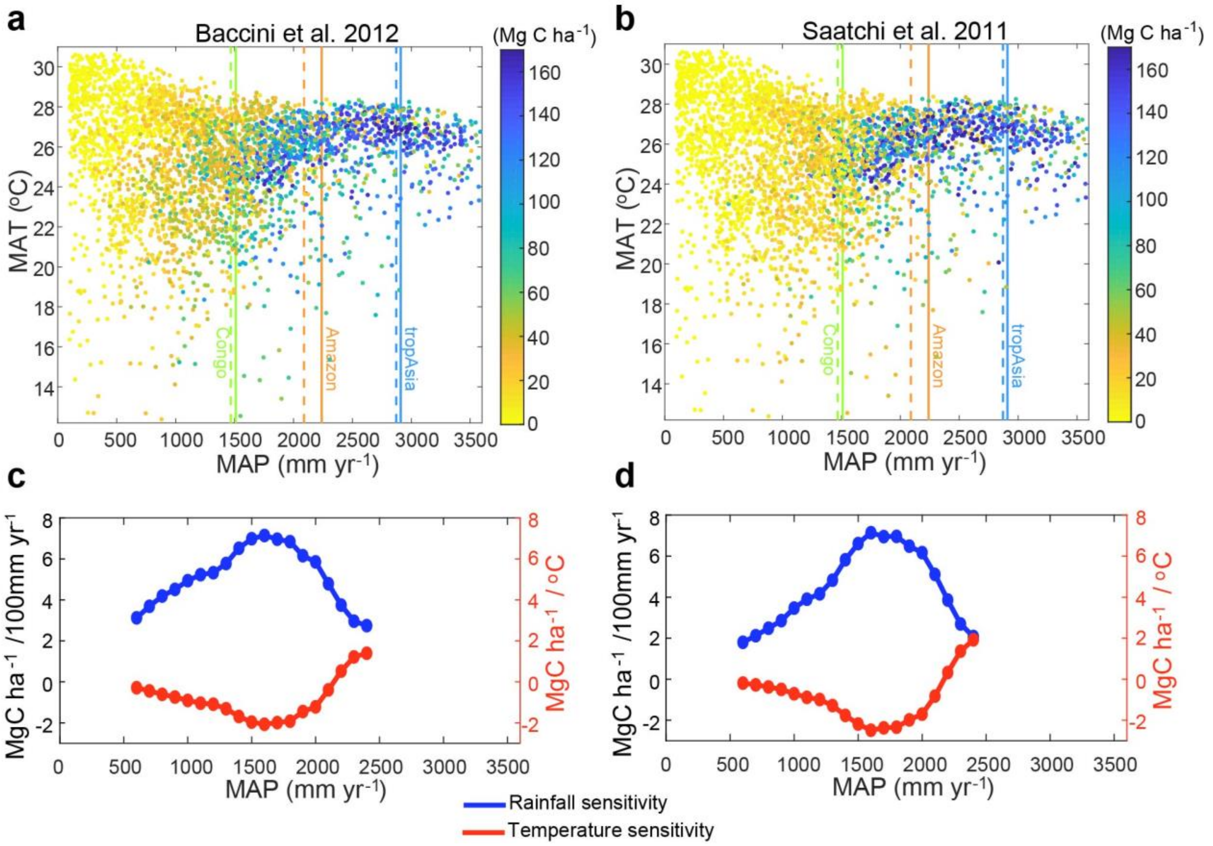

Supplementary Figure 6 | Same as Fig. 2a,c but using different aboveground biomass (AGB) data from ref. [1] and ref. [2].

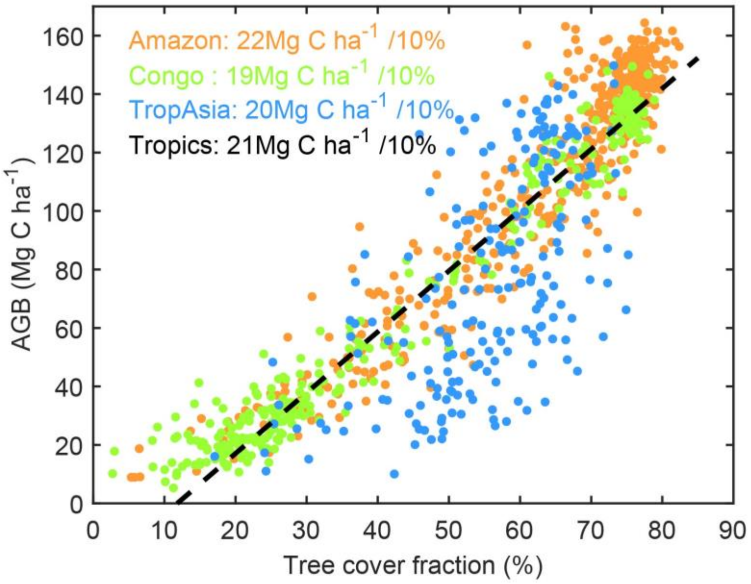

Supplementary Figure 7 | Observational relationship between the aboveground biomass (AGB) and tree cover fraction in the tropics. AGB was derived from the ESA–CCI BIOMASS project for the year of 2017 (see data descriptions in Methods). The tree cover fraction is derived from MODIS Vegetation Continuous Field (VCF, MOD44B) during the year of 2017 (see data descriptions in Methods).

Supplementary Table 1 | Impacts of idealized deforestation on latent heat flux, sensible heat flux and surface net radiation in the tropics.

Region	Latent heat flux (W m^{-2})	Sensible heat flux (W m^{-2})	Surface net Radiation* (W m^{-2})
Amazon	−6.9 (3.5)	2.3 (2.5)	−4.6 (2.5)
Congo	−3.4 (4.5)	0.1 (2.1)	−3.3 (2.9)
Tropical Asia	−2.1 (3.8)	0.7 (2.5)	−1.4 (1.9)

*Surface net radiation is computed as the sum of latent heat and sensible heat flux assuming the ground heat flux being negligible at the annual time scale.

Supplementary Table 2 | A list of regression parameters for the spatial variations in observation–based aboveground biomass (AGB) related to climate variables (see Methods), including MAP (mm yr^{-1}), P_{amp} (mm $month^{-1}$), PRD (mm $month^{-1}$), MAT (°C), T_{amp} (°C), MAXT (°C), and VPD (hPa).

**Model (AGB=)**	**a**	**b**	**R^2**	**RMSE**
a*MAP+b*MAT+ε	0.034	−0.316	0.49	32.1
a*MAP+b*T_{amp}+ε	0.026	−1.148	0.26	40.4
a*MAP+b*MAXT+ε	0.030	−0.280	0.60	29.8
a*MAP+b*VPD+ε	0.026	−0.676	0.25	40.5
a*P_{amp}+b*MAT+ε	0.136	0.105	0.19	40.3
a*P_{amp}+b*T_{amp}+ε	0.210	−3.707	0.12	44.1
a*P_{amp}+b*MAXT+ε	0.106	0.427	0.01	46.7
a*P_{amp}+b*VPD+ε	0.193	−1.469	0.00	46.9
a*PRD+b*MAT+ε	0.510	0.888	0.39	34.8
a*PRD+b*T_{amp}+ε	0.395	1.173	0.06	45.6
a*PRD+b*MAXT+ε	0.393	0.637	0.57	30.9
a*PRD+b*VPD+ε	0.372	0.858	0.07	45.2

Supplementary Table 3 | A list of regression parameters for the spatial variations in model–based aboveground biomass (AGB) related to climate variables (see Methods), including MAP (mm yr^{-1}), P_{amp} (mm $month^{-1}$), PRD (mm $month^{-1}$), MAT (ºC), T_{amp} (ºC), MAXT (ºC), and VPD (hPa).

Model (AGB=)	a	b	R^2	RMSE
a*MAP+b*MAT+ε	0.031	-0.041	0.60	23.0
a*MAP+b*T_{amp}+ε	0.033	-0.595	0.62	22.7
a*MAP+b*MAXT+ε	0.032	-0.056	0.61	23.0
a*MAP+b*VPD+ε	0.033	-0.453	0.62	22.5
a*P_{amp}+b*MAT+ε	0.166	-0.142	0.26	31.5
a*P_{amp}+b*T_{amp}+ε	0.206	-2.595	0.40	28.4
a*P_{amp}+b*MAXT+ε	0.176	-0.250	0.27	31.3
a*P_{amp}+b*VPD+ε	0.191	-1.345	0.36	29.2
a*PRD+b*MAT+ε	0.413	1.340	0.33	29.8
a*PRD+b*T_{amp}+ε	0.587	2.496	0.04	35.9
a*PRD+b*MAXT+ε	0.431	1.092	0.31	30.3
a*PRD+b*VPD+ε	0.600	1.409	-0.04	37.4

Supplementary Table 4 | Cumulative loss in primary forest fraction and the biophysical aboveground biomass (AGB) losses during 1850–2015.

	**Mean primary forest loss (%)**	**LUMIP forest loss (%)**	**LUMIP rainfall change (%)**	**LUMIP temperature change (ºC)**	**Rainfall climatology (mm yr^{-1})**	**Rainfall sensitivity (Mg C ha^{-1} / 100 mm yr^{-1})**	**Temperature sensitivity (Mg C ha^{-1} / ºC)**	**Area (km^{-2})**	**Biophysical AGB loss (Tg C)**
Amazon	−11.5	−44.7	−6.7	0.5	2240	3.4	0.34	5.84×10^{6}	−741*
Congo	−8.4	−38.7	−2.7	0.1	1510	5.7	−1.78	3.68×10^{6}	−200
Tropical Asia	−22.9	−31.2	−1.3	−0.1	2910	0.0	0.0	4.81×10^{6}	0

*This value was computed by the equation: {[(−11.5%) ÷ (−44.7%) × (−6.7%) × 2240 mm yr^{-1} × (3.4 Mg C ha^{-1}/100 mm yr^{-1})] + [(−11.5%) ÷ (−44.7%) × 0.5 ºC × (0.34 Mg C ha^{-1}/ºC)]} × (10^{-6} × 100 Tg C km^{-2} / Mg C ha^{-1}) × 5.84×10^{6} km^{-2} ≈ −741 Tg C

Supplementary References

1. Saatchi, S. S. et al. Benchmark map of forest carbon stocks in tropical regions across three continents. *Proc. Natl. Acad. Sci. U.S.A.* **108**, 9899-9904 (2011).
2. Baccini, A. et al. Estimated carbon dioxide emissions from tropical deforestation improved by carbon-density maps. *Nat. Clim. Chang* **2**, 182-185 (2012).
